# Effects of Interrupted Daily Routine Due to COVID-19 on Circadian Chronotype and Leisure Time Physical Activity

**DOI:** 10.3390/sports10070109

**Published:** 2022-07-14

**Authors:** Justine M. Renziehausen, David H. Fukuda

**Affiliations:** Institute of Exercise Physiology and Rehabilitation Science, School of Kinesiology and Physical Therapy, College of Health Professions and Sciences, University of Central Florida, Orlando, FL 32816, USA; justine.renziehausen@ucf.edu

**Keywords:** circadian chronotype, COVID-19, physical activity, circadian rhythm

## Abstract

Circadian chronotype is dependent on many factors including age, physical activity participation, eating and sleeping patterns, and typical schedule. Recently, the COVID-19 pandemic resulted in schedule changes for most individuals. Therefore, the purpose of this study is to examine whether sport participation influences circadian chronotype and physical activity and whether COVID-19 restrictions have impacted chronotype scores. Briefly, 128 physically active males *(n* = 62) and females (*n* = 66) between 18 and 55 years old (24.7 ± 7.1) completed a survey consisting of demographics information, the Morningness–Eveningness Questionnaire (MEQ), and the Godin Leisure Time Physical Activity Scale (LTPA). Participants were asked to answer relevant questions about their habits/preferences before and after COVID-19-related restrictions were implemented. MEQ scores categorized individuals into morning (MT), intermediate (IT), and evening (ET) chronotypes. Three-way (pre-COVID-19 chronotype x sport participation x time) repeated measures ANOVA was conducted to evaluate differences in MEQ and LTPA. A significant main effect of time was found for MEQ (*p* = 0.018) and LTPA (*p* = 0.002), indicating changes following COVID-19. A significant time x chronotype interaction was shown for MEQ (*p* < 0.001) with MT (*p* < 0.001), IT (*p* = 0.044), and ET (*p* = 0.044) individuals indicating chronotype-specific changes following COVID-19. LTPA was decreased and MEQ scores changed following COVID-19, with shifts toward IT scores.

## 1. Introduction

The circadian rhythm is an internal 24 h cycle regulated via the suprachiasmatic nucleus (SCN) in the hypothalamus. Most biological systems in the body exhibit a diurnal pattern [1]. For example, blood pressure, body temperature, and numerous hormone secretion patterns follow 24 h cycles. The SCN ensures the synchronization of all systems and their “clocks” to maintain homeostasis [1], which is crucial for overall health and optimal performance. This alignment, however, is individualized, where some individuals peak earlier in the day than others. This is referred to as circadian chronotype [1,2].

There are three main chronotypes: morning-type (MT), neither- or intermediate-type (IT), and evening-type (ET). Chronotypes are characterized by an individual’s preferred time of day. MT individuals wake earlier, exercise earlier, and generally feel their best earlier in the day than IT or ET individuals [1]. Studies have shown that questionnaires pertaining to preferred times to sleep, eat, and perform physically and mentally fatiguing tasks correspond to physiological markers of the circadian rhythm, such as core body temperature and melatonin secretion [3]. Additionally, it has been shown that individuals who work outside of their preferred times compromise their health. One study demonstrated that nurses that were scheduled for hours during non-optimal times of day increased their risk of developing type II diabetes [4].

Additionally, research has shown that athletes perform better during their optimal time of day. Previous research found that MT marathon runners recorded better race times when the race began earlier in the day, as opposed to a midday start [5]. Similarly, another study determined that MT collegiate rowers performed significantly better in the morning than the evening, while ET rowers performed the same at either time of day [6]. Other research has demonstrated that performance during a cardiovascular endurance test may differ up to 26% for each chronotype based on time of day [7]. Further investigations revealed significant differences in maximal voluntary contractions performed at different times of day based on chronotypes, where MT performed best in the afternoon (14:00 h) and ET performed best in the evening (20:00 h) [8].

Time of day preference may be dependent on a variety of factors, including aging, participation in physical activity, and schedule changes [9,10]. Previous research has indicated that more females tend to be MT than males, particularly those over the age of 40 [10]. It has also been suggested that elite athletes tend to be MT, while less than 10% of this population identifies as ET [5,9]. This is unusual, as studies in the general population typically report that at least 25% of individuals identify as ET [11]. Although studies have suggested that the competitive level of an athlete may influence chronotype, research has been predominantly conducted with high-level aerobic athletes. While the existing exercise-related chronotype research has focused primarily on high-level aerobic athletes, limited data are available examining recreationally active non-athletes and those engaged in a variety of activities.

Lastly, the circadian rhythm of individuals is composed of endogenous (i.e., age and gender) and exogenous (i.e., light stimulus) factors. Most exogenous factors can be retrained to adapt to changes in schedules that may occur [10,12]. Recently, numerous individuals experienced changes to their schedule when restrictions were implemented for safety purposes during the COVID-19 pandemic [13]. Furthermore, individuals who reported consistent resistance training before COVID-19 indicated that gym closures caused exercise habits to change [14]. Specifically, participants reported exerting less effort during exercise routines and perceived less effectiveness of each workout [14]. Sleep habits were reportedly altered during this time as well, as individuals were going to sleep later, staying asleep for less time, and napping more throughout the day [15]. Although the circadian rhythm can adapt to environmental changes, less is known about the shifting of chronotype scores.

Understanding individual chronotypes may assist in scheduling training and competitions for both health purposes and optimal performance. Therefore, the primary purpose of this study was to determine whether sport participation influences circadian chronotype and leisure time physical activity. A secondary purpose was to determine whether any changes to chronotype have occurred due to schedule changes related to COVID-19. It is hypothesized that greater levels of physical activity correspond to higher chronotype scores (i.e., MT) and that schedule changes related to COVID-19 impact chronotype scores.

## 2. Materials and Methods

### 2.1. Study Design

This study followed a cross-sectional survey design to examine circadian chronotypes in active individuals. Chronotypes, exercise training activities, and schedule changes following COVID-19-related restrictions were recorded. Participants were recruited from the university and social media websites (e.g., Facebook, Instagram, and others) via flyers. To be included in this study, participants were required to have been physically active at least 3 days a week for 6 or more months prior to schedule changes related to COVID-19. Participants were excluded from data analysis if they did not participate in physical activity at least 3 days a week prior to COVID-19-related restrictions. After receiving a written explanation of all procedures involved in participation, consent was obtained. All procedures were approved by the University of Central Florida’s Institutional Review Board.

### 2.2. Participants

The final analyses included 128 active males (*n* = 62) and females (*n* = 66) between the ages of 18 and 55 years old (24.7 ± 7.1 years). All participants were located in the United States. A total of 166 individuals were recruited. Of these, 16 did not meet the inclusion criteria and were excluded. An additional 21 did not complete the survey and were excluded from all analyses. Descriptive statistics are displayed in Table 1.

### 2.3. Measures and Procedures

Participants were asked to complete a survey via Qualtrics. Data were collected between July 2020 and August 2020. This survey consisted of three parts: demographics questionnaire, Morningness–Eveningness Questionnaire (MEQ), and Godin’s Leisure Time Physical Activity Scale (LTPA) (further described below). When relevant, participants were asked to consider questions under two conditions: prior to and after COVID-19. These questions appeared side by side and were labeled “PRIOR to any schedule changes due to COVID-19” and “AFTER any schedule changes due to COVID-19”. This survey took approximately 20 min to complete.

#### 2.3.1. Demographics

Participants were asked to complete a demographics questionnaire consisting of 20 multiple-choice items pertaining to age, gender, race/ethnicity, physical activity participation, and schedule. Participants were asked to confirm that they had been active for at least 3 days a week for a minimum of 6 months prior to COVID-19-related restrictions to be included in the study. Participants were additionally asked if they participated in an organized sport to determine the nature of their exercise engagement; those who answered “no” were categorized as recreationally active.

#### 2.3.2. Morningness–Eveningness Questionnaire

This questionnaire consists of 19 items pertaining to preferred schedules [2]. For example, one question asked, “During the first half-hour after you wake up in the morning, how tired do you feel?” Other items evaluated preferred time to sleep, exercise, and perform mentally fatiguing tasks. Responses were multiple choice scales of 4–6 choices. Individuals are categorized according to the total sum of their responses as follows: morning-type (59–86), neither-type (42–58), and evening-type (16–41). Previous research has determined this questionnaire to have sufficient internal consistency (Cronbach’s alpha = 0.837) in a similar population (men and women over the age of 25 (38.4 ± 12.4)) [16]. Additionally, test-retest reliability was determined to be acceptable (Cronbach’s alpha = 0.84) [17].

#### 2.3.3. Godin Leisure Time Physical Activity Questionnaire

This questionnaire consists of 3 items pertaining to weekly activity and intensity of exercise [18]. The questionnaire asked, “During a typical 7-day period, how many times on average do you do the following kinds of exercise for more than 15 min during your free time?” Exercise activities were categorized as strenuous (e.g., running), moderate (e.g., fast walking), or mild (e.g., yoga), and the weekly frequencies of each category were tabulated. Frequency of activity and the associated metabolic equivalent of task (i.e., 9× frequency of strenuous activity, 5× frequency of moderate activity, and 3× frequency of mild activity) were summed to obtain the total score. Individuals were then categorized as active (24 or more total units), moderately active (14–23 units), or insufficiently active (fewer than 14 units).

### 2.4. Statistical Analysis

Three-way [chronotype (MT, IT, ET) × sport participation (recreational, organized) × time (pre-COVID, post-COVID)] repeated measures ANOVAs were conducted to evaluate MEQ scores and LTPA. Bonferroni post-hoc analyses were conducted when appropriate. Cohen’s d values were used to evaluate effect size and interpreted as follows: *d* = 0.2 indicates a small effect, *d* = 0.5 indicates a medium effect, and *d* = 0.8 indicates a large effect. A Wilcoxon signed-rank *t*-test was conducted to determine differences in employment in response to COVID-19. Chi-Square analyses were conducted to determine differences in exercise times and work-shift start times for the overall sample and chronotype groups before and after COVID-19. Follow-up evaluations were examined using confidence intervals based on independent binomial distributions. One participant who reported being in the 6:00 p.m.–12:00 a.m. category for work-shift start time after COVID-19 was excluded from the MT analysis due to no MT participants being included in this category prior to COVID-19-related restrictions. Repeated measures ANOVAs were conducted to evaluate the effect of employment (e.g., employment status stayed the same or job was lost) on MEQ and LTPA. Pearson correlations were conducted to examine the relationship between age, LTPA change scores, and MEQ change scores (post/pre values). All statistical procedures were conducted using JASP (version 13, Amsterdam, the Netherlands) with a significance level set at α < 0.05.

## 3. Results

No differences were shown for MEQ (F = 0.745; *p* = 0.390; *d* = 0.076) or LTPA (F = 0.214; *p* = 0.645; d = −0.041) scores between recreationally active individuals and those engaged in organized sport. A significant correlation was found between LTPA and MEQ change scores (r = 0.301, *p* ≤ 0.001). No significant correlations were noted between age and either LTPA change scores (r = 0.116, *p* = 0.193) or MEQ change scores (r = 0.098, *p* = 0.273). Differences in MEQ and LTPA by sport participation and chronotypes can be found in Table 2.

**Figure 1:**
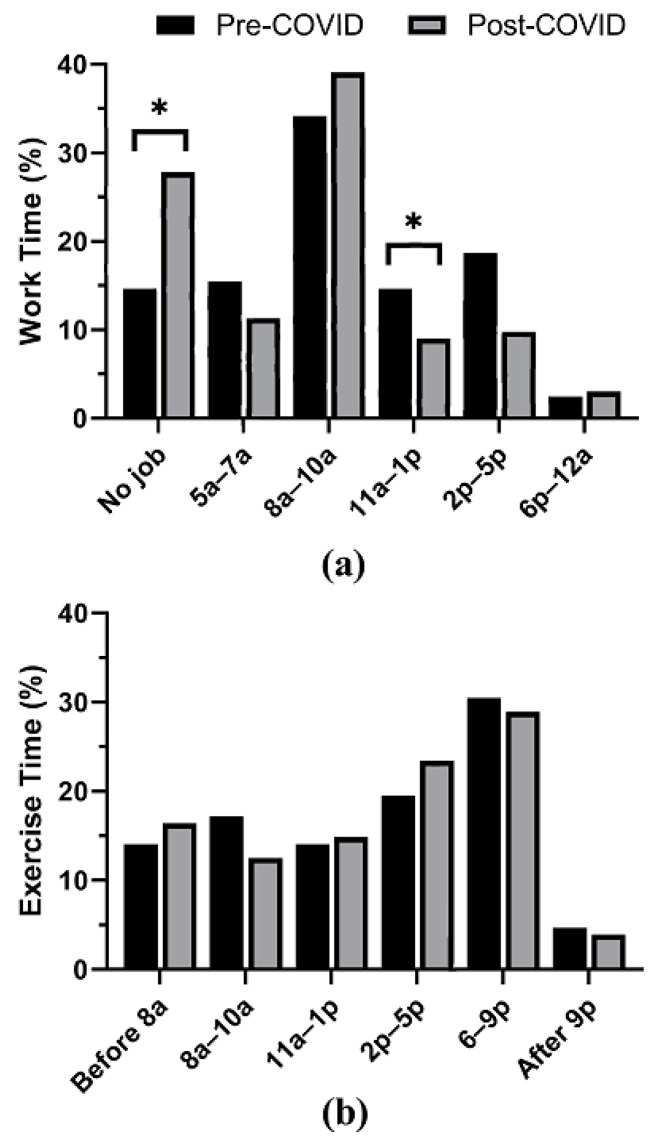
(**a**) Work-shift start times before and after COVID-19-related restrictions. (**b**) Exercise times before and after COVID-19-related restrictions. * indicates potential changes using confidence intervals based on independent binomial distributions.

A significant difference in employment following COVID-19 (W = 77.5, *p* < 0.001) was observed. No significant interactions between MEQ and employment (F (1, 121) = 1.146, *p* = 0.286) or LTPA and employment (F (1,121) = 0.004, *p* = 0.951) were observed. Five participants gained a job during this time and were excluded from this analysis. No significant differences in exercise times were noted before and after COVID-19 for the overall sample [X^2^ (5, N = 128) = 3.46, *p* = 0.629] or any of the chronotypes: MT [X^2^ (5, N = 36) = 3.27, *p* = 0.659], IT [X^2^ (5, N = 73) = 3.93, *p* = 0.560], or ET [X^2^ (5, N = 19) = 6.2, *p* = 0.287]. Significant differences in work-shift start times were found before and after COVID-19 for the overall sample [X^2^ (5, N = 128) = 30.419, *p* < 0.001] and the IT chronotype [X^2^ (5, N = 73) = 34.45, *p* < 0.001], while there was no significant difference for MT [X^2^ (4, N = 36) = 6.04, *p* = 0.196] or ET [X^2^ (4, N = 19) = 2.25, *p* = 0.690]. Confidence intervals indicated potential changes in the proportion of individuals with work-shift start times during the 11:00 a.m.–1:00 p.m. time period for the overall sample and for those with a change in employment status for the overall sample and the IT chronotype. Data from exercise times and work shift start times for the overall sample are shown in Figure 1.

A significant main effect of time was found for both MEQ (F = 15.773, *p* = 0.018) and LTPA (F = 15.773, *p* = 0.002), indicating changes in response to COVID-19 of −2.053 units (95% confidence interval = −0.361 to −3.744 units; d = −0.212) and −8.533 units (95% confidence interval = −3.328 to −13.378 units; d = −0.287), respectively.

**Figure 2:**
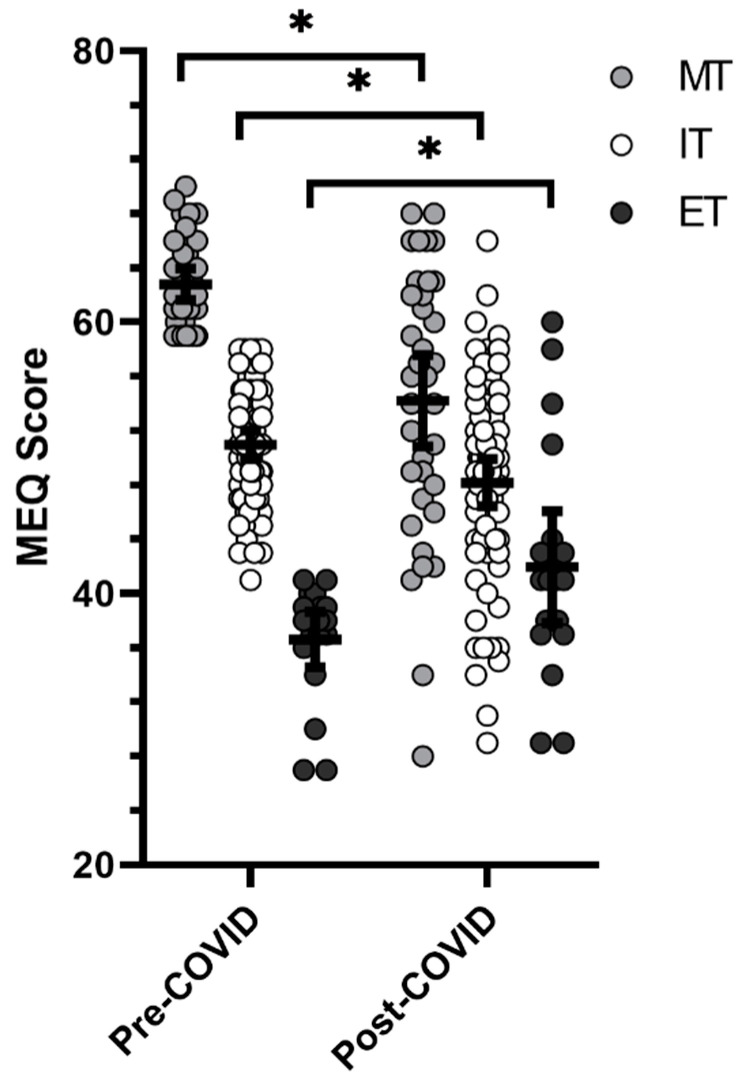
Shifts in chronotype scores before and after COVID-19-related restrictions. * significant differences from pre- to post-COVID-19 responses (*p* < 0.05).

A significant time × chronotype interaction was shown for MEQ (F = 16.395, *p* < 0.001), with MT individuals indicating changes in response to COVID-19 of −8.557 units (95% confidence interval = −4.392 to −12.722 units; *p* < 0.001; d = −0.871), IT individuals indicating changes of −2.315 units (95% confidence interval = −0.675 to −5.306 units; *p* = 0.044; d = −0.444), and ET individuals indicating changes of +4.714 units (95% confidence interval = −10.423 to −0.995 units; *p* = 0.044; d = 0.530). Chronotype shifts before and after COVID-19 are shown in Figure 2.

## 4. Discussion

The purpose of this study was to determine whether sport participation influences circadian chronotype and leisure time physical activity. It was also to examine changes in chronotype scores and leisure time physical activity following safety measures taken in response to COVID-19. It was hypothesized that (1) sport participation and level of physical activity would affect MEQ chronotype scores and (2) that there would be changes in chronotype scores and leisure time physical activity before and after the COVID-19-related restrictions. The results of this study indicate that both MEQ chronotype scores and LTPA scores were significantly different before and after COVID-19-related restrictions. Additionally, the results indicated that there is no significant difference in MEQ chronotype scores based on sport participation or level of physical activity. This may be due to several factors, described further below.

The recent literature has discussed several routine changes in response to COVID-19 [13]. According to one study, individuals have reported differences in eating, sleeping, social, and exercise habits [13]. For example, participants were asked what time they wake up in the morning before and after COVID-19. Responses indicated that before COVID-19, only 26 (12%) participants woke up at 8:00 a.m. or later, compared to 94 (42%) participants after COVID-19. Changes in exercise habits were also noted, with 42% of participants exercising 3 or more times per week before COVID-19, compared with 21% after COVID-19 [13]. Similarly, another study reported significant changes in sleep behavior, including later wake times and bedtimes, increased daytime naps, and poorer sleep quality overall [15]. Additionally, 55% of participants reported working from home as a result of the COVID-19-related restrictions [15], which likely impacted their daily routines, such as eliminating the need to commute to and from work [19]. Avoiding a morning commute may allow individuals to wake later and increase sleep duration. A recent study found that participants work significantly later (about 1 h) following COVID-19 restrictions. This may be especially advantageous for intermediate- and evening-type chronotypes, as they are able to adjust their sleep–wake cycle to better fit their biological clocks and potentially avoid social jetlag [19]. Furthermore, a study examining the effects of COVID-19 restrictions on chronotypes and sleep determined that the magnitude of change was dependent on changes to participants’ work schedules [20]. In the present study, participants reported significant changes to work-shift start times. These schedule changes, as well as sleep and exercise routine changes, have the potential to impact circadian rhythm, and it is possible that these factors played a role in the MEQ and LTPA score changes seen in the present study.

Further analyses were conducted to elucidate the relationship between variation in MEQ and LTPA during the examined time period. The previous literature has suggested that age impacts sleep characteristics (e.g., sleep onset and sleep duration) and indicates that younger participants were more greatly impacted by the effects of COVID-19-related restrictions [20]. However, age was not significantly correlated to either MEQ or LTPA scores in the present study. This may be due to the sample in this study, which consisted predominantly of students, and therefore older individuals were underrepresented. Additionally, it was determined that MEQ and LTPA change scores were significantly positively correlated. This suggests that although exercise times were not statistically different in the present study, exercise frequency and chronotypes may have a relationship such that a greater change in physical activity corresponds to a greater change in chronotype score. This is in line with previous research that indicates correlations between amount of physical activity and chronotypes [21,22]. For example, one study reported associations between ET scores and low leisure time physical activity [21].

In addition, the previous literature has observed differences in chronotypes for elite endurance and team sport athletes [9,23]. The present study aimed to examine differences in chronotype scores based on sport participation, (i.e., whether participants were involved in an organized sport or not). While results of the present study indicate that there were no differences in chronotype scores based on sport participation, this may be due to the inclusion of various levels of active individuals. Because research has indicated that elite athletes who participate in lengthy competitions and frequent training sessions show differences in chronotypes compared to the general population [5,9,24], it was hypothesized that higher participation in physical activity would result in differences in MEQ scores. Results showed no significant differences in MEQ score based on level of physical activity. It is important to note that previous research has been conducted in predominantly endurance athletes [5,9], while the present study included a wider variety of exercise activities indicative of more generalized training habits, which may have contributed to these results.

There are strengths in the present study that should be noted. This study is one of the first to examine changes in MEQ scores and LTPA before and after COVID-19. Furthermore, there is limited research that examines differences in MEQ scores based on varying levels of exercise engagement and physical activity. There were also limitations to this study. First, the sample size may not have been large enough to draw conclusions. Furthermore, more recreationally active individuals (n = 84) participated in this study than those who participated in organized sports (n = 44), which may have influenced MEQ and LTPA results. Additionally, some of the participants involved in organized sports reported playing at the club level and may not have been at competitive levels sufficient enough to demonstrate differences in chronotypes [24]. Second, to meet the inclusion criteria, participants were required to have been exercising for 3 or more days per week. Excluding individuals who were sedentary prior to COVID-19 may have influenced data in terms of the range of LTPA scores and their influence on MEQ scores. Only self-reported data on physical activity were able to be collected for this study, which may lead to under- or over-reporting of activity levels. Online data collection and recruiting (via social media and university-related platforms) may have impacted the sample and the results obtained as participants needed to have access to the internet. Additionally, this study was collected retrospectively, and participants were asked to recall habits from a previous time period. Lastly, this study did not examine sleep duration or quality, which may impact chronotype and activity levels.

## 5. Conclusions

In conclusion, the results of this study indicate significant changes in chronotype scores based on the MEQ, with an overall shift towards IT. This may have implications for work schedules and favors implementing strategies that allow employees to work within their optimal time frame to potentially increase productivity. Additionally, there was decrease in leisure time physical activity following COVID-19 restrictions, and there were no significant differences in MEQ scores based on sport participation or level of physical activity. Future research should consider recruiting a larger sample overall, as well as a larger sample of competitive athletes and inactive individuals for comparison purposes. Future research should also aim to examine potential differences in chronotypes between recreationally active and sedentary individuals.

## Figures and Tables

**Table 1:** Sample demographics (n = 128).

Variable	*N* (%)
Race	Caucasian/White—69 (54%)
	African American/Black—18 (14%)
	Hispanic/Latino—37 (29%) Asian—9 (7%) Native American/Alaska Native—2 (2%) Other—3 (2%)
MEQ	Before COVID-19—52.17 ± 9.28 MT—36 (28%), IT—73 (57%), ET—19 (21%)
	After COVID-19—48.95 ± 9.18 MT—20 (16%), IT—81 (63%), ET—27 (21%)
Organized Sport Participation	44 (34%)
Employed	Before COVID-19—111 (87%)
	After COVID-19—91 (71%)
Student	109 (85%)


**Table 2:** Morningness-Eveningness Questionnaire scores and Godin Leisure Time Physical Activity Scale scores (mean ± SD) by chronotype and sport participation prior to COVID-19-related restrictions.

		MT pre	MT Post	IT pre	IT Post	ET pre	ET Post	All pre	All Post
		n = 36		n = 73		n = 19		n = 128	
MEQ	Recreational	63.3 ± 3.4	54.7 ± 8.6	51.2 ± 4.8	47.5 ± 7.4	36.7 ± 4.4	43.7 ± 9.2	52.4 ± 9.4	48.9 ± 8.7
	Organized Sport	61.9 ± 3.4	53.5 ± 12.3	50.5 ± 3.9	49.6 ± 7.5	36.6 ± 4.4	39.0 ± 7.0	51.7 ± 9.1	49.1 ± 10.1
	All	62.8 ± 3.4	54.2 * ± 9.9	51.0 ± 4.5	48.2 * ± 7.4	36.6 ± 4.2	42.0 * ± 8.6	52.2 ± 9.3	49.0 * ± 9.2
LTPA	Recreational	51.0 ± 17.8	42.1 ± 20.2	46.7 ± 19.6	40.4 ± 23.7	45.8 ± 14.3	41.7 ± 23.4	47.8 ± 19.4	41.0 ± 22.5
	Organized Sport	54.0 ± 25.2	42.8 ± 19.3	55.1 ± 22.4	41.5 ± 26.6	45.9 ± 16.5	38.9 ± 23.3	53.3 ± 22.3	41.5 ± 23.7
	All	52.1 ± 20.5	42.3 ± 19.6	49.5 ± 20.8	40.8 ± 24.5	45.8 ± 14.7	40.6 ± 22.8	49.7 ± 19.9	41.2 * ± 22.8

* Significantly different from pre (*p* < 0.05).

## Data Availability

Not applicable.
